# Lazarus Phenomenon: Return of Spontaneous Circulation After Cessation of Prolonged Cardiopulmonary Resuscitation in a Patient With COVID-19

**DOI:** 10.7759/cureus.17089

**Published:** 2021-08-11

**Authors:** Maria Cristina Martinez-Ávila, Amilkar Almanza Hurtado, Alvano Trespalacios Sierra, Tómas Rodriguez Yanez, Carmelo Dueñas-Castell

**Affiliations:** 1 Department of Epidemiology and Clinical Research Unit, Nuevo Hospital Bocagrande, Cartagena, COL; 2 Intensive Care Unit, Gestión Salud IPS, Cartagena, COL

**Keywords:** cardiopulmonary resuscitation, critical care, lazarus phenomenon, self-resuscitation, covid-19, sars-cov-2

## Abstract

The pandemic caused by the SARS-CoV-2 or COVID-19 infection has had an unimaginable impact on health systems worldwide. Cardiorespiratory arrest remains a potentially reversible medical emergency that requires the performance of a set of maneuvers designed to replace and restore spontaneous breathing and circulation. Suspending cardiopulmonary resuscitation (CPR) usually corresponds to an ethical-clinical dilemma that the health professional in charge must assume. The “Lazarus phenomenon” is an unusual syndrome with a difficult pathophysiological explanation, defined as the spontaneous return of circulation in the absence of any life support technique or after the cessation of failed CPR maneuvers.

We present the case of a 79-year-old patient hospitalized in the intensive care unit for septic shock of pulmonary origin associated with COVID-19 infection who presented cardiorespiratory arrest that required unsuccessful resuscitation maneuvers for 40 minutes, declared deceased. After 20 minutes of death, he presented a return to spontaneous circulation.

The pathophysiological changes of the Lazarus phenomenon remind us of the limitations we have in determining when to end cardiopulmonary resuscitation and that its interruption must be approached with more caution, especially in the context of the COVID-19 pandemic.

## Introduction

The pandemic caused by the SARS-CoV-2 or COVID-19 infection has had an unimaginable impact on healthcare systems worldwide, generating modifications in human lifestyles and in common medical care protocols. To date, on July 25, 2021, 194,080,019 confirmed cases and 4,162,304 deaths have been reported in the world. In Colombia, 4,716,798 cases and 118,538 deaths have been reported [1]. Cardiorespiratory arrest remains a potentially reversible medical emergency that requires the performance of a set of maneuvers aimed at replacing and restoring spontaneous breathing and circulation known as cardiopulmonary resuscitation (CPR) [2]. When faced with a positive COVID-19 patient, the following questions arise naturally: What do we do if a positive COVID-19 patient has a cardiorespiratory arrest and CPR must be started? For how long do we do it, considering their prognosis and the scarcity of resources?

Suspending CPR usually corresponds to an ethical-clinical dilemma that the health professional must assume [3]. According to the provisional guide for basic and advanced life support in adults, children, and neonates with suspected or confirmed COVID-19 [3], having a person with an arrest rhythm for a time greater than 10 minutes receiving support measures, with protected airway and no return to spontaneous circulation is reason enough to stop CPR and declare death. However, considering this context, even when the exact numbers and outcomes of CPR related to COVID-19 are unknown, cessation of therapeutic efforts is a difficult decision to make.

The "Lazarus phenomenon" refers to the famous biblical figure whom Jesus resurrected.

Then Jesus shouted: "Lazarus, get out of there!" And the dead man came out of the grave.

(John 11: 43-4)

This unusual syndrome with a difficult pathophysiological explanation is defined as the spontaneous return of circulation, either in the absence of any life support technique or after the cessation of failed CPR maneuvers [4]. The resulting pathophysiologic changes in this phenomenon serve as a reminder of our limitations in determining when to end cardiopulmonary resuscitation; therefore, its interruption should be approached more carefully.

## Case presentation

**Figure 1:**
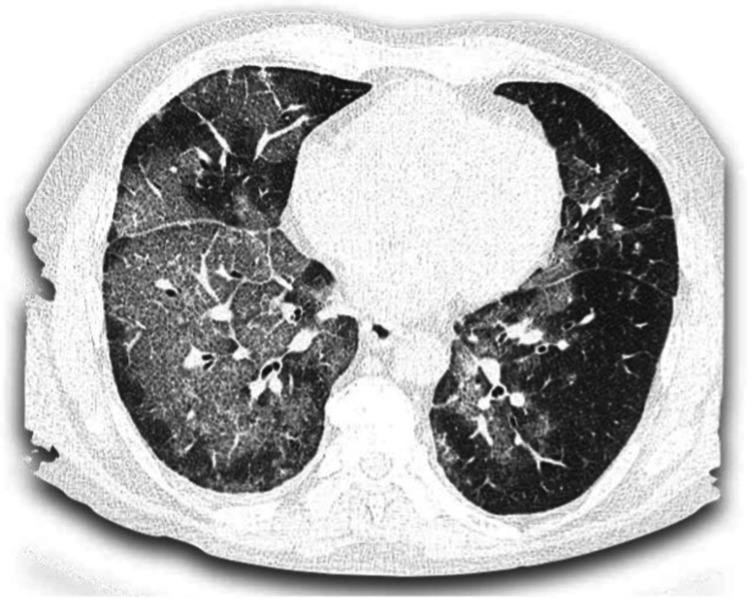
Axial nonenhanced chest CT images (lung window) show bilateral multiple ground-glass opacities

A 79-year-old female patient with a history of arterial hypertension was admitted to the emergency department due to five-day history of unquantified fever peaks, asthenia, and adynamia that worsened accompanied by respiratory distress. Upon admission, she was in poor general condition, dehydrated, mean arterial blood pressure 59mmHg, heart rate 110bpm, respiratory rate 30rpm, saturating 77% with high-flow oxygen, presenting subcostal draws. Initial blood samples (Table 1) show leukocytosis, neutrophilia, lymphopenia, high potassium, increased creatinine, blood urea nitrogen (BUN), and lactate dehydrogenase (LDH); arterial gases in acid-base balance with severe hypoxemia (partial pressure of carbon dioxide/fraction of inspired oxygen [PaO2/FiO2] ratio - PaFi 56.9), and chest tomography with involvement of 75% of the lung parenchyma with ground glass pattern (Figure 1). Endotracheal intubation was performed. The patient was transferred to the COVID-19 intensive care unit. She presented clinical-radiological suspicion of SARS-CoV-2 infection and on bacterial superinfection with septic shock and associated acute kidney injury. Real-time polymerase chain reaction (RT-PCR) COVID-19, markers of prognosis and severity were requested; broad-spectrum antibiotic coverage was started, after taking blood-cultures, intravenous corticosteroids according to available evidence, antiparasitic due to the risk of larval migration while using systemic steroids, gastroprotection, and thromboprophylaxis was also assessed

**Table 1:** Patient's blood test BUN: blood urea nitrogen; LDH Lactate dehydrogenase; CPR: cardiopulmonary resuscitation; PCO2: partial pressure of carbon dioxide; HCO3: bicarbonate; PaFi: PaO2/FiO2 ratio; PaO2: partial pressure of oxygen; FiO2: fraction of inspired oxygen

Test	Initial results	With CPR	Reference value
Leukocytes (m/mm^3^)	27080		4000-11000
Neutrophils	98% (26504)		40-60%
Lymphocytes	2.11% (574)		15-45%
Hemoglobin (g/dL)	12.5		12-15
Platelets (m/mm^3^)	158000		150000 - 450000
Potassium (mEq/L)	6.5	7.4	3.5-5.0
Sodium (mEq/L)	138	129	135-145
Creatinine (mg/dL)	3.54	5.1	0.5-1.4
BUN (mg/dL)	86	195	9.2-30
LDH (U/L)	1020	1637	<300
Ferritin (ng/mL)	450	1474	<250
Arterial Blood Gases	pH 7.38 pCo2 34mmHg HCO3 24mEq/L PaFi 56.9	pH 6.48 pCo2 207.6mmHG pO2 5.1mmHg HCO3 3.5mEq/L PaFi 11.7	pH 7.35-7.45 pCo2 35-45mm Hg HCO3 22-26mEq/L PaFi >300

**Figure 2:**
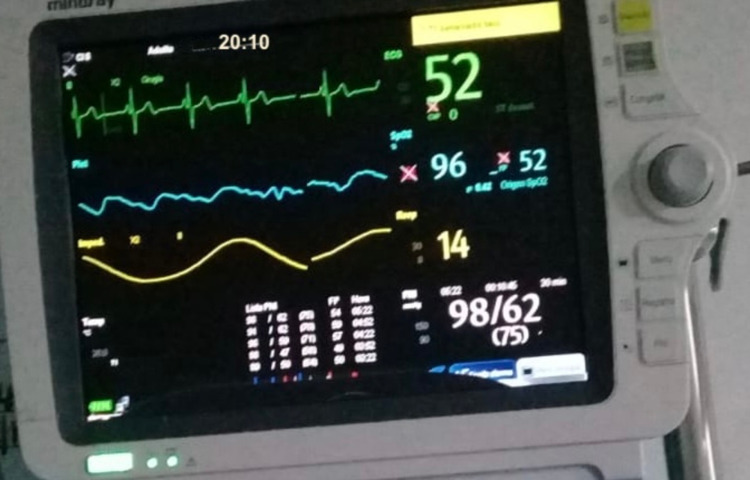
Bradycardia

**Figure 3:**
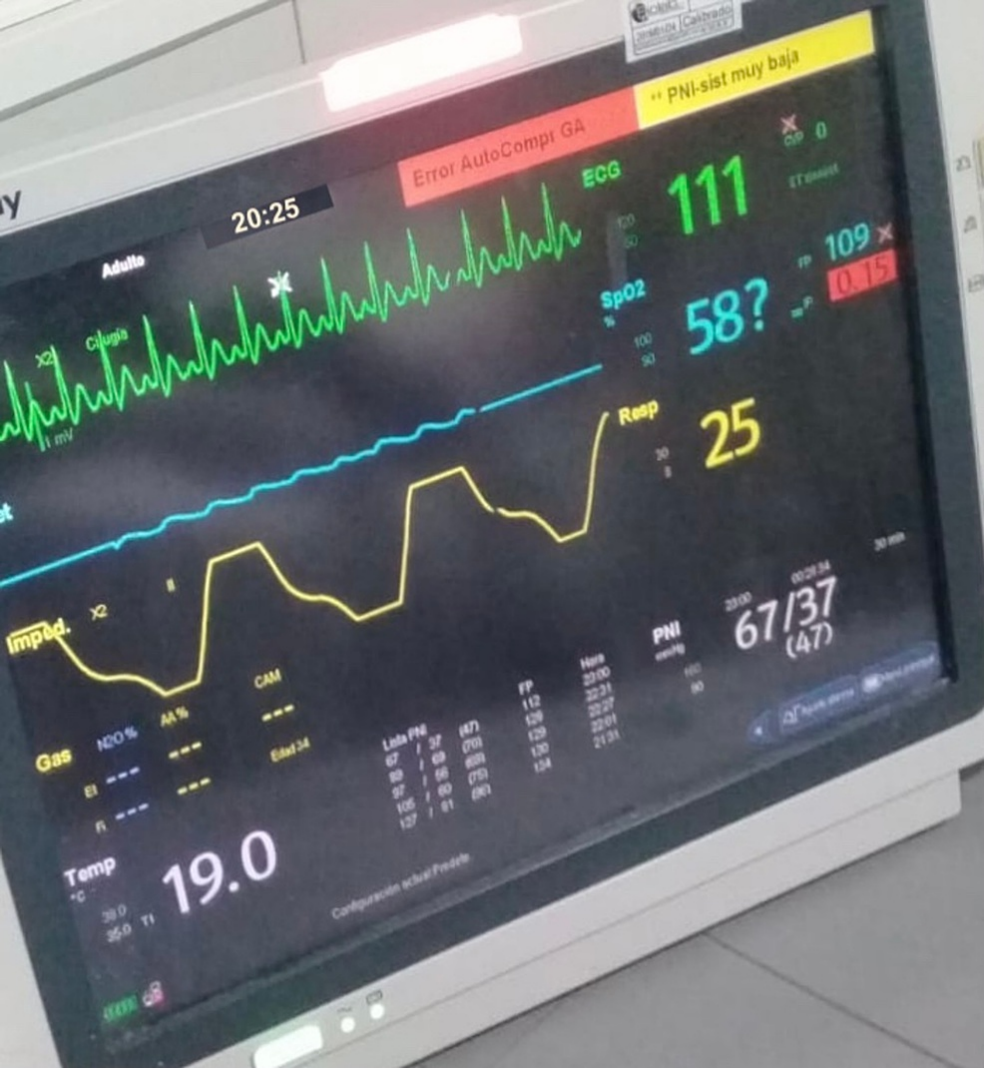
Return of spontaneous circulation

**Figure 4:**
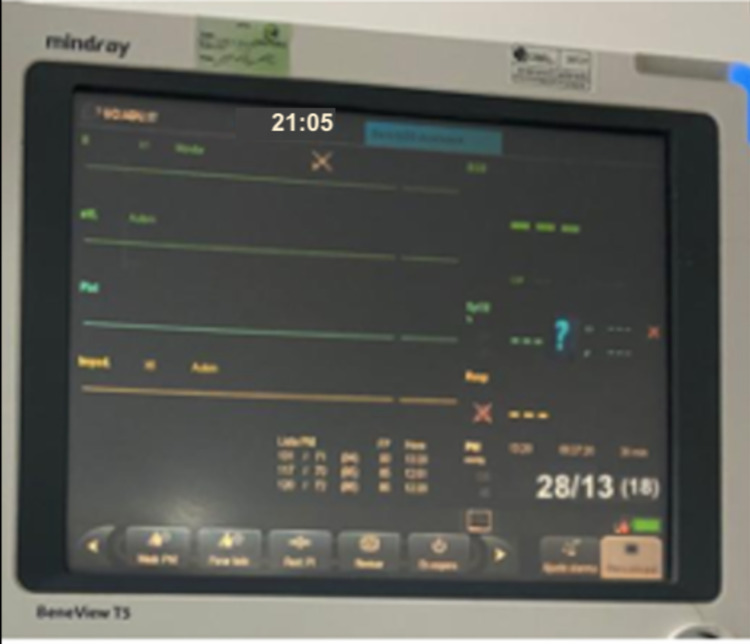
Asystole

During this time, staying in the intensive care unit, COVID-19 infection was confirmed by RT-PCR. The patient presented torpid evolution with persistence of severe hypoxemic respiratory failure despite invasive mechanical ventilation with neuromuscular blockers, dual sedation (midazlolam/fentanyl IV infusion), and prone position, with the requirement of double vasoactive support (norepinephrine/vasopressin IV infusion). She presented mixed acidosis and hyperlactacidemia, oligoanuria, with BUN/creatinine dissociation, prolonged clotting times, altered biomarkers of severity, and was febrile. On the seventh day of care, extreme bradycardia (Figure 2) progressing to asystole was evidenced, noting the absence of a pulse, isoelectric line in the monitor, cardiac arrest was identified and blue code was activated. CPR maneuvers were initiated with protected contact according to the COVID-19 protocol with the return of spontaneous circulation (ROSC) after 10 minutes of resuscitation (Figure 3), for which reason it was indicated to begin a third inotropic drug in infusion. After 15 minutes of the post-resuscitation state, the patient returned to a sequence of multiple non-shockable arrest rhythms that required continuous CPR again. After 40 minutes of assistance and in the final rhythm of asystole (Figure 4), the ventilator was still giving O2 in assisted control mode, end-tidal CO2 (ETCO2) was 6mmHg, her temperature was 32ºC, new blood tests were assessed (Table 1), the resuscitation maneuvers were stopped - not only due to temperature but also due to a set of clinical and paraclinical criteria - and death was finally declared.

**Figure 5:**
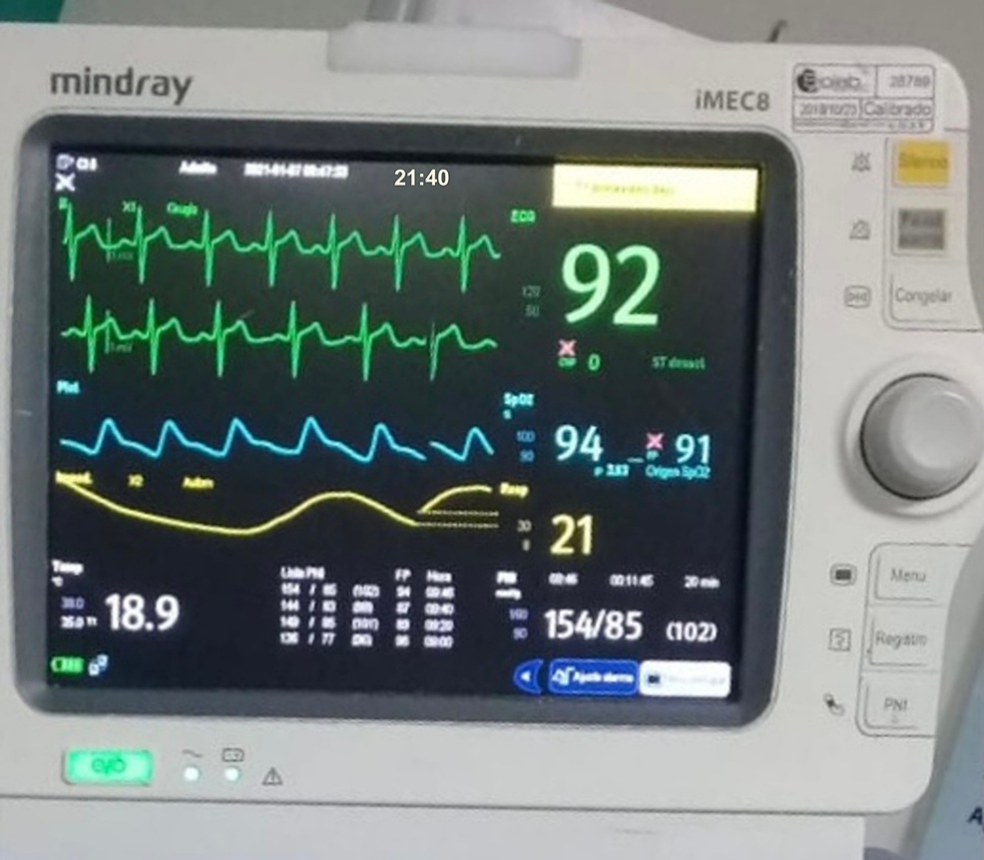
Lazarus phenomenon

According to the guidelines of the Colombian Ministry of Health regarding the handling of corpses due to the COVID-19 pandemic, it is established that the corpse must be kept intact and limit its manipulation, without removing catheters, probes, or tubes that may contain the fluids from the corpse [5]. The patient continued with vascular accesses, orotracheal tube, and urinary catheter. Twenty minutes after the death was declared, while waiting for the sheets to be shrouded and cotton to cover the natural orifices, the patient spontaneously recovered her pulse and respiration, evidenced by thoracic expansion; nevertheless, regaining of consciousness was not confirmed. The monitors were connected showing a heart rate of 92bpm, blood pressure 154/85mmHg, oxygen saturation 94% with adequate coupling to the ventilator (Figure 5), pupils responded to light. Blood samples were requested, arterial blood gas analysis and post-cardiac arrest care were started. Finally, she died 21 days later from multiple organ dysfunction syndrome septic shock because of viral pneumonia confirmed by COVID-19. 

## Discussion

The Lazarus phenomenon is an exceptional pathology of difficult explanation and even cataloged as anecdotal, which was first reported in the literature in 1982; however, it was not until 1993 that the anesthesiologist Jack Bray coined the term [6], which comes from the word Eleazaros, which means in Hebrew "God has helped," alluding to the biblical passage where Jesus after four days of death raised Lazarus. Although it is a little-known syndrome that can be disconcerting to the healthcare provider, it is more common than people might think. In recent decades, the medical literature and the media have revealed more than 60 cases of self-resuscitation or ROSC after cessation of CPR maneuvers or in the absence of vital supports [7-9]. The low publication rate of clinical cases may be associated with fears of the health personnel related to ethical-legal actions, disbelief of the rest of the health care personnel before what happened, and professional discredit [10-11]. Also because delayed ROSC could lead to questions being asked about whether resuscitation had been conducted properly and whether it was stopped prematurely [10-11]. 

The pathophysiological mechanism that produces the late return of spontaneous circulation is still unknown. It is even considered to be multifactorial, possibly in combination. Among the various theories raised are acidosis and electrolyte disorders such as hyperkalemia thanks to the persistence of potassium at the intracellular level causing the myocardium to retract for long periods of time [12], temporary asystole after defibrillation, pharmacological delayed action of the drugs used during CPR such as adrenaline [13-14]. Cardiovascular factors include: coronary perfusion pressure (CPP) as low as 15mmHg can produce a return of spontaneous circulation after asystole; intrinsic vasomotor function of capacitance and resistance blood vessels may maintain CPP so that even when resuscitation has ceased, CPP may be high enough to restart the heart [7]. Return of myocardial function following termination of resuscitation (TOR): myocardial reperfusion due to spontaneous dislodging of endovascular plaque from a coronary artery [7]. 

Another hypothesis raised is associated with poorly controlled ventilation techniques such as the process of dynamic hyperinflation of the lungs for excess tidal volumes or rapid increase in positive pressure without adequate time for exhalation in artificial ventilation, generating hemodynamic involvement [15]. In other words, the increase in intrathoracic pressure may have led to air trapping with increased pressure at the end of expiration, known as auto-PEEP (auto-positive end-expiratory pressure), which led to a significant impedance to venous return, affecting cardiac output, leading to cardiac arrest [16]. Therefore, it has been suggested that autoresuscitation is due to the medical interventions that were performed during resuscitation, but their effectiveness was delayed for some reason.

We consider that according to the clinical history of the patient whose main condition was respiratory etiology, this hypothesis is the most accurate. Once CPR and supplemental artificial invasive mechanical ventilation were suspended, chest pressures at the level of the respiratory system gradually normalized, allowing ROSC and cardiac pulse reappearance. In the case of our patient, it is a plausible explanation, since the patient had high volume requirements considering the persistence of severe hypoxemic respiratory failure and associated with it presented a non-shockable arrest rhythm compatible with what is described in the literature. Having received 100% oxygen delivery during CPR there may have been enough oxygen reserve in the residual volume of the lungs to support their oxygen demands during the unassisted period. It is advisable to use protective mechanical ventilation whenever possible to avoid the appearance of auto-PEEP and affect systemic circulation.

At this point, you might ask, when or why to stop CPR? In the context of COVID-19, the risk to the clinical team is increased and resources can be profoundly more limited [3,17]. Therefore, some considerations were taken into account to continuing resuscitation as: age, 79-year-old patient, comorbidities: arterial hypertension, and severity of illness: septic shock in conjunction with the probability of success against the risk to rescuers to suspend CPR. It is noteworthy that, therapeutic hypothermia has been widely implemented, its benefits are still questioned, and several issues remain unanswered, including the optimal time to initiate cooling. Experimental research has shown that intra-arrest therapeutic hypothermia (IATH) increases the success rate of defibrillation attempts in ventricular fibrillation and has beneficial effects on cardiac function, including improved left ventricular function and reduced myocardial infarction size [18]. However, other studies have shown that intra-arrest hypothermia after delayed cardiopulmonary resuscitation did not improve survival [19]. On the other hand, this patient had already had multiple continuous cardiac arrests that lasted more than 40 minutes with the impossibility of achieving an ETCO2 greater than 10mmHg by waveform capnography in a previously intubated patient, which means poor prognosis and survival after short term [3]. All of the above analyzed in an emergency situation under a multimodal approach and in an epidemiological context of care such as the COVID-19 pandemic were sufficient reasons to decide to end resuscitation efforts.

Regarding the time interval from TOR/diagnosis of death to ROSC, in a systematic review where 38 bibliographic sources were analyzed, they reported that it varied between “a few seconds” and a maximum of 33 minutes. It is worth mentioning that the expression "a few seconds" was interpreted as a time less than 10 minutes; however, in one case described, the patient was found alive in the morgue and in seven cases the information on the time elapsed was not available [9].

There is no evidence available that links the underlying pathology of the patient with the appearance of the Lazarus phenomenon. The cause of cardiac arrest in COVID-19 patients is related to the acute respiratory distress syndrome that leads to refractory hypoxemia as well as major cardiovascular complications caused by cytokine storm or myocardial dysfunction on behalf of the direct effect of the coronavirus on the heart [20]. The survival prognosis in CPR in COVID-19 patients is unknown [17]. The National Academies of Sciences, Engineering and Medicine of the United States affirms that the standards of care in case of crises such as COVID-19 infection are aimed at "saving as many lives as possible" under severe resource limitations; maintaining the fundamental ethical principles: "justice, care, do no harm, reasonable use of resources, transparency in decision-making, coherence, proportionality and accountability". Therefore, once a blue code is established, CPR should be started waiting for ROSC. 

There is substandard information in the literature regarding post-CPR outcomes in COVID-19. It was identified that for patients with confirmed SARS-CoV-2 infection in New York, United States, the median CPR time was 14 minutes, 42% of the patients survived a first cardiac arrest, however, none survived to hospital discharge and survival time to death was 2.8 hours [21]. While in Wuhan, China only 13.2% of patients had ROSC and 2.94% survived for at least 30 days and only one patient achieved a favorable neurological outcome at 30 days [22]. In our experience, 58% of patients have survived cardiac arrest, but overall survival rates remain low.

## Conclusions

It is important that health personnel delivering resuscitation are aware of the existence of the Lazarus phenomenon before being confronted with it. Considering the appearance of a Lazarus phenomenon, death should not be certified after the immediate cessation of CPR, especially in the following conditions: immediately after defibrillation, if vasopressor infusions and/or mechanical ventilation are used; in order to avoid prematurely TOR before therapeutic measures could have an adequate effect. The need to continue non-invasive hemodynamic monitoring for a reasonable time of at least 10 minutes, if not more, after stopping CPR is essential to verify the persistence of asystole prior to certifying death. Check for and correct all reversible causes of cardiac arrest before considering TOR. Take into careful consideration how long CPR has been employed before TOR and declaring death. Likewise, the absence of hemispheric functions and the brain stem must be confirmed to declare brain death. Some authors recommend, as lung hyperinflation is one of the most accepted and studied theories of this phenomenon, disconnecting the ventilator as a last effort in patients who do not respond to resuscitation efforts; however, it is at the discretion of the treating physician.

This case report experience emphasizes that resuscitation should be terminated with caution. More investigation is needed to be done in order to establish some criteria to reliably determine when a Lazarus phenomenon occurs. Nevertheless, the possibility of autoresuscitation should not affect the decision about when to terminate resuscitation.
